# The prevalence of hepatitis B antigen-positivity in the general population of Mashhad, Iran

**Published:** 2011-05-01

**Authors:** Farhad Fathimoghaddam, Mohammad Reza Hedayati-Moghaddam, Hamid Reza Bidkhori, Sanaz Ahmadi, Hamid Reza Sima

**Affiliations:** 1 Research Center for HIV/AIDS, HTLV and Viral Hepatitis, Iranian Academic Center for Education, Culture and Research (ACECR), Mashhad Branch, Mashhad, IR Iran; 2 Division of Gastroenterology, Department of Internal Medicine, Imam Reza Hospital, School of Medicine, Mashhad University of Medical Sciences, Mashhad, IR Iran

**Keywords:** HBsAg, Hepatitis B virus, Population group, Iran, Risk factor

## Abstract

**Background:**

Hepatitis B virus (HBV) infection is a significant health problem throughout the world.

**Objectives:**

We aimed to evaluate the prevalence of hepatitis B antigen (HBsAg) seropositivity in the general population of Mashhad, northeast of Iran.

**Patients and Methods:**

One thousand six hundred fifty-two healthy individuals aged 1 to 90 (Mean; 29.1 ± 18.5) from all 12 municipalities of Mashad were selected randomly by multistage cluster sampling. Informed consent was obtained, and demographics and medical histories were collected. Twice-reactive samples were considered HBsAg-positive by ELISA. Chi-square test and logistic regression were applied to analyze the factors related to HBsAg positivity using SPSS 16.0.

**Results:**

The overall prevalence of HBsAg positivity was 1.39% (95% CI, 0.91% to 2.12%); 2.0% and 0.89% among men and women, respectively (p = 0.054). Infection was more prevalent in older (p = 0.019) and married persons (p = 0.001), Afghanis (p = 0.046), and those with a history of traditional cupping (p = 0.005). There was no association between HBV infection and gender; literacy; income; employment; family size; or history of blood transfusion, dental procedure, surgery, hospitalization, or tattooing. By logistic regression analysis, age was the only variable that had a significant association with infection (p = 0.026).

**Conclusion:**

It seems that the prevalence of HBV infection in Mashhad is slightly lower than that of the nation.

## Background

HBV infection is a major global public health problem. It is estimated that between 350 to 400 million people are hepatitis B virus (HBV) carriers in the world, approximately 1 million of whom die from HBV-related liver diseases annually [1][2]. The prevalence of HBV infection differs throughout the world. There are low-prevalence areas, such as the United States, Canada, western Europe, Australia, and New Zealand, in which the prevalence is 0.1% to 2%; intermediate-prevalence areas, such as Mediterranean countries, Japan, central Asia, the Middle East, and Latin and South America (2% to 8%); and high-prevalence areas, such as southern Asia, China, and sub-Saharan Africa (8% to 20%) [3][4].

The Islamic Republic of Iran (Iran) is an intermediate endemicity area regarding the prevalence of HBV in the general population [5]. Initial studies have estimated that more than 50% of Iranian patients with cirrhosis and about half of those with hepatocellular carcinoma are HBV carriers [6]. Further, up to 3.9% of Iranians are HBsAg-positive [7]. Similarly, Farzadegan et al. (1979) reported an incidence of HBsAg of 3.4% and 8.4% among voluntary and professional blood donors, respectively [8].

Conversely, recent studies have demonstrated that the prevalence of HBV infection among the general population and blood donors in Iran has declined [5][9]. A systematic review in 2008 estimated the prevalence of HBV infection in Iran to be 2.14% - 2.55% and 2.03% among males and females, respectively [9]. It has been suggested that the observed decline is due to the inclusion of neonates in the national HBV vaccination program since 1993 and the vaccination of high-risk groups against HBV [10][11][12]. Also, a nationwide study by the Iranian Blood Transfusion Organization (IBTO) stated that the incidence of HBsAg among voluntary blood donors declined signiﬁcantly from 1.8% in 1998 to 0.4% in 2007 [5], which could be explained by the blood safety measures that were implemented by the IBTO across the country, such as improved donor selection and exclusion of replacement donations [5]. The modes of transmission for HBV include perinatal infection, unprotected sexual contact, intravenous drug use, and blood transfusion. The chief route of transmission differs between regions. In high-prevalence areas, perinatal infection is the major route [13]. However, in intermediate-prevalence areas, horizontal transmission, especially in early childhood, causes most chronic HBV infections, and unprotected sexual intercourse and intravenous drug use in adults are the principal transmission routes in low-prevalence areas [14].

Mashhad, the center of Khorasan-e-Razavi province, in the northeast of Iran, is its second largest city, with a population of 2.4 million. It attracts about 20 million pilgrims annually as an important holy city. There have been few studies regarding the prevalence of HBV infection in the general population of this area. A cross-sectional study on healthy population in the range of 2-100 years of Khorasan province in 1998 calculated an HBsAg-positive rate of 3.6% [15]. Ghanaat, et al. tested 1500 individuals who underwent an STD examination in Mashhad between 1998 and 2000, determining a seroprevalence of HBsAg in 12.2% and 14.3% of 90 persons with gonorrhea and 70 with syphilis, respectively [16]. Also, per Khorasan-e-Razavi Blood Transfusion Organization reports, 1.2% of donors who were admitted to blood transfusion centers during 2001 to 2005 were HBsAg-positive [17].

## Objectives

The present study was conducted to determine the prevalence of hepatitis B antigen and its related factors in the general population of Mashhad, Iran.

## Materials and Methods

In this cross-sectional study, 1678 apparently healthy residents of Mashhad were selected by multistage cluster sampling from all 12 municipalities between May and September 2009. Pilgrims and high-risk groups, such as hemodialysis patients, were not included in this survey. These 12 areas included 40 districts, and each district had many subdivisions. First, we estimated the number of households that was proportional to the total population in every district. Next, one subdivision was selected randomly from every district, and some blocks were chosen randomly in every subdivision. Next, the total number of households in each selected block was estimated, and at least 20 households were chosen in each block by systematic random sampling method. Finally, 1 person was selected in each household, and we tried to include equal numbers of the both genders; we also established percentiles by age according to the 2006 census (less than 6 years, 6-11, 12-16, 17-20, 21-24, 25-29, 30-34, 35-44, 45-54, and 55 and older). The study was approved by the Research Deputyship of the Iranian Academic Center for Education, Culture & Research (ACECR) regarding methodological and ethical issues. The study purpose was clearly discussed with the participants, and informed consent was obtained (for children, the informed consent was obtained from their parents), and demographic characteristics and medical histories, including history of blood transfusion, dental procedures, surgery, hospitalization, and traditional cupping, were collected using a questionnaire.

Five-milliliter venous blood samples were obtained from each individual. Serum was separated by centrifugation, and samples were stored at -20°C. Serum samples were screened for HBsAg by ELISA using the EIAgen HBsAg Kit (Adaltis Italia S.p.A., Italy) per the manufacturer's instructions. Twice-reactive samples by ELISA were considered HBsAg-positive. Data were expressed as mean, standard deviation, and percentages and were analyzed by chi-square test using SPSS 16.0. Logistic regression was used to analyze the factors related to HBsAg positivity. A p < 0.05 was considered statistically significant.

## Results

One thousand six hundred seventy-eight individuals, ranging from 1 to 90 years, were enrolled; 763 (45.5%) were male and 915 (54.5%) were female, and the mean age was 27.9 ± 19.0 and 30.0 ± 18.1 years, respectively. Twenty-six persons refused to have blood taken and were excluded from subsequent analyses. In the screen of 1652 serum samples by ELISA, 23 (1.39%) (15 men and 8 women) were positive for HBsAg (95% CI, 0.91% to 2.12%). No significant relation were observed between genders and positivity of HBsAg (p = 0.054) (Table 1).

**Table 1:** Sociodemographic factors related to HBsAg positivity in the general population of Mashhad, Iran

**Variable**	**No.**	**Positive **(%)	**P-value**
**Sex**			0.054
**Male**	749	2.00	
**Female**	903	0.89	
**Age** (years) a			0.019
**< 6**	129	0.78	
**6-11**	155	0.00	
**12-16**	157	0.00	
**17-20**	183	0.55	
**21-24**	180	0.00	
**25-29**	179	2.23	
**30-34**	132	0.76	
**35-44**	190	3.16	
**45-54**	161	2.48	
**≥ 55**	186	3.23	
**Marital status** (people older than 14)			0.001
**Single**	416	0.48	
**Married**	774	1.94	
**Divorced/widowed**	57	7.02	
**Ethnic background**			0.046
**Iranian**	1576	1.21	
**Afghani**	61	4.92	
**Literacy **(people older than 6)			0.43
**Illiterate**	110	1.82	
**Primary-secondary school **(1-8 years)	628	1.59	
**High school** (9-12 years)	495	1.82	
**Academic**	265	0.38	
**Household monthly income** (million Rials^b^)			0.39
**< 3**	799	1.63	
**3-5**	544	1.65	
**> 5**	224	0.45	
**Employment** (people older than 14)			0.059
**Unemployed**	677	2.44	
**Employed**	489	1.03	
**Family Size**			0.20
**1-2**	213	2.82	
**3-4**	707	1.13	
**≥ 5**	635	1.42	

^a^ Ten percentiles for age according to the 2006 census

^b^ 1 million Rials is equal to about 100 USD.

HBV infection was significantly associated with age. HBsAg-positive individuals were considerably older than others (42.7 ± 18.2 vs. 28.9 ± 18.4 years, respectively, p < 0.001). The prevalence of HBV infection was 0.26% in people under 15, 0.24% in 15-24-year olds, 1.61% in 25-34-year olds, and 2.98% in those above 35 years (p = 0.003). In individuals under 25 years, HBsAg was detected in 2 persons-a 4-year-old Afghani child born in Isfahan, Iran, with a history of 3 HBV vaccinations, and an 18-year-old single man who was born in Mashhad, without a history of HBV immunization.

By univariate analysis, HBV infection was also associated with being married (p = 0.001), being Afghani (p = 0.046), and having history of traditional cupping (Hijamat) (p = 0.005) (Table 1 and Table 2). There was no association between HBV infection and literacy, family size, monthly income, employment, or a history of blood transfusion, surgery, hospitalization, dental procedure, and tattooing. Binary logistic regression analysis was used to estimate the potential risk factors of infection. The variables in the equation showed in Table 3 were age (< 25 vs. 25 years and above), marital status (single vs. married at least once), ethnicity (Iranian vs. Afghani), and a history of traditional cupping (no vs. yes). Being older than 25 years was the only factor that predicted the risk of infection (p = 0.026).

**Table 2:** Medical factors related to HBsAg positivity in the general population of Mashhad, Iran

**Variable**	**No.**	**Positive **(%)	**p-value**
**Transfusion**			0.24
**Yes**	63	3.17	
**No**	1543	1.36	
**Hospitalization**			0.74
**Yes**	626	1.28	
**No**	1013	1.48	
**Surgery**			0.57
**Yes**	551	1.63	
**No**	1089	1.29	
**Dentistry procedure**			0.37
**Yes**	348	2.59	
**No**	1278	1.10	
**Tattooing**			0.17
**Yes**	53	3.77	
**No**	1573	1.34	
**Traditional cupping**			0.005
**Yes**	233	3.43	
**No**	1398	1.07	

**Table 3:** Logistic regression analysis of the association of HBsAg positivity with selected variables (Mashhad, Iran, 2009)

**Variable**	**p-value**	**OR a**	**95% CI b**
**Age **	0.026	9.80	1.31-73.46
**Marital Status**	0.527	1.68	0.34-8.43
**Ethnic background**	0.098	3.60	0.79-16.38
**Traditional cupping**	0.476	1.43	0.54-3.80

^a^ OR: Odds Ratio

^b^ CI: Confidence Interval

## Discussion

HBV infection is considered a major public health problem in the world, involving many people in many countries [1][2]. Iran is located in the Middle East, which is known as an area with an intermediate to high prevalence of HBV infection; however, there are differences between countries in this region [4]. Bahrain and Kuwait are areas with low endemicity, with HBsAg carrier rates under 2%. These countries have included hepatitis vaccination as a part of their Expanded Program on Immunization, covering over 80% of the population. Countries with intermediate endemicities in this region include Cyprus, Iraq, and the United Arab Emirates, with carrier rates of 2% to 5% in their general population. Areas of high endemicity include Egypt, Jordan, Oman, Palestine, Yemen, and Saudi Arabia, with carrier rates of 2% to 18.5% in their general population [18].

Studies have indicated that the prevalence of HBV infection differs among the general population between various parts of Iran. In the present study, we found that the overall HBsAg positivity in the general population of Mashhad, located in Khorasan-e-Razavi province, was 1.39%, which is lower than the national prevalence (2.14%) [9]; however ,the confidence intervals in mentioned studies overlap. Abdolahi, et al. reported the highest prevalence of HBsAg among the general population of Golestan province, 8.86%, which was equivalent to high-endemicity areas [9]. Also, the prevalence of HBsAg in the general population of Golestan (city of Gonbad), Hormozgan, and Hamedan (city of Nahavand) provinces is 4.3%, 2.4%, and 2.3%, respectively, which are equivalent to rates in intermediate endemicity areas [9][20][21].

Conversely, Nokhodian, et al. [22] and Bayat-Makou, et al. [23] reported 1.3% and 1.2% prevalence rates for HBsAg in the general population of Isfahan and Azarbayejan (city of Tabriz) provinces, respectively, which are equal to those in low endemicity areas. We noted higher rates of HBsAg positivity in the general population than in blood donors in Mashhad [17]. This finding is expected because the high-risk group was screened prior to blood donation.

We have also noted that the prevalence of HBV has decreased dramatically in the last decade in Iran, which now renders it an area with low endemicity [12]. Several factors could contribute to the decrease in HBV infection in Iran and in this study. The most important factor could be the implementation of a national vaccination program in 1993 for all neonates as a part of an Expanded Program on Immunization (EPI). The coverage rate of this vaccination program has reached 99%, according to national data from 2009 [24]. Also the immunization of high-risk groups, such as health care workers and blood product recipients, as recommended by national vaccination program, could be another important factor in the reduction of HBsAg positivity [12][25]. Consistent with many studies in Iran [11][22][26], our study showed that the prevalence of HBV infection is significantly higher among older people. The very low prevalence of HBsAg among people aged younger than 17 years demonstrates that the national vaccination program for neonates is an effective method of reducing infections. Moreover, the 3-round HBV vaccination campaigns were implemented for 17-year-olds in 2007 [27], meaning that individuals aged older than 17 years at the time of this study had no received active immunization against HBV through the national vaccination programs for neonates and adolescents. Nevertheless, such programs might also play an important role in the very low prevalence of HBV infection among those aged older than 17 years, probably due to the reduction in intrafamilial transmission. The results of 2 large-scale national seroepidemiological surveys in Iran showed a significant decline in the seropositivity rate in the 2-14 but not 15-69-year-olds between 1991-1999 [11]. HBV carrier rates fell from 1.3% in 1991, 2 years before the launch of the national HBV vaccination program, to 0.8%, 6 years after the launch in 1999 in this age group. Increases in people's knowledge about HBV risk factors through the media and educational programs for students could also affect the prevalence of infection, especially among youth. On the other hand, the increased possibility of intrafamilial transmission in older individuals could explain the age-related trend of infection. We found that HBV infection was more prevalent in men than women, although this difference was not significant. This finding has been confirmed in previous studies [11][26], whereas others do not support it [20][22].

Our study also noted a higher prevalence of HBsAg among married people versus singles. In Iran, the average age at first marriage is 26.2 and 23.2 years for men and women, respectively [28]. Thus, most married people have not been included in the national vaccination programs and are not protected against the infection. Yet, sexual contact is one of the common transmission routes for HBV infection, and the probability of acquiring the infection from an HBsAg-positive person's sexual partner is 4% to 15% [9]. Compared with western countries, premarital sexual relationships in the Iranian community are expected to be rare due to special cultural and religious backgrounds [29], meaning that sexual transmission of HBV infection between spouses might be a reason for the higher prevalence of infection among married people. Finally, our study observed a higher prevalence of HBsAg among people of Afghani ethnicity. Quddus, et al. reported an 8.3% rate of HBsAg positivity among Afghan refugees in a camp in Balochistan, Pakistan [30]. Thus, it would be valuable to monitor HBV infections among foreign immigrants in cities with large immigrant populations, such as Mashhad.
